# Understanding teaching and learning conceptions among clinical faculty as a means to improve postgraduate training

**DOI:** 10.5116/ijme.5f2a.76eb

**Published:** 2020-08-28

**Authors:** Jaime L. Pacifico, Jeroen Donkers, Johanna Jacobs, Cees van der Vleuten, Sylvia Heeneman

**Affiliations:** 1 De La Salle University College of Medicine, De La Salle Medical Health Sciences Institute, The Philippines; 2 Maastricht University, The Netherlands; 3 University Medical Center, Utrecht, The Netherlands

**Keywords:** Postgraduate, clinical teachers, conceptions, teaching, learning

## Abstract

**Objectives:**

This study aimed to determine the conceptions of teaching and learning of postgraduate medical teachers.

**Methods:**

We invited postgraduate clinical teachers to fill out COLT (Conceptions on Learning and Teaching) questionnaire, an 18-item instrument designed to measure the conceptions of faculty in undergraduate medical education, and did a confirmatory factor analysis (CFA) to test if it was valid to be used in a postgraduate situation. Cluster analysis was done to determine different teacher profiles. We subsequently did a qualitative study among 12 clinical teachers to further explore issues related to conceptions of teaching. We used a semi-structured interview guide with vignettes summarizing five perspectives of teaching.

**Results:**

Four criteria of goodness of fit indices were met, although six items had to be removed from the original COLT items. Three clusters were identified, and 51% of participants favored a transmission teaching-style perspective. For the qualitative part, three themes were identified. Majority of the teachers preferred apprenticeship and nurturing teaching-style perspective, even if they were educated through a transmission teaching-style perspective.

**Conclusions:**

Our study has shown that the COLT, although initially designed for undergraduate medical setting in the Netherlands was a valid tool in a different setting and population, with some modifications. Both the survey and the interview studies showed that the majority of the faculty chose the transmission perspective initially, but when introduced to the other perspectives, preferred apprenticeship and nurturing. The faculty readily embraced other perspectives of teaching that they believe to take into consideration the well-being of the trainees.

## Introduction

Any educational system involves interaction between teachers, learners and learning materials.^1^ It has been shown that both teachers and learners will approach teaching and learning in different ways, indicating multiple beliefs or conceptions about these educational processes.^2^ For teachers, this will influence how they teach and assess.^1^ For the use of the word 'conceptions', commonly the definition by Pratt is used: "conceptions are specific meanings attached to phenomena which then mediate our response to situations involving those phenomena… In effect, we view the world through the lenses of our conceptions, interpreting and acting in accordance with our understanding of the world".^3^ Pratt^3^ posits that this relation between conceptions and behavior impacts on how educators think about teaching. In later publications, Pratt has used conceptions and perspectives interchangeably: "A perspective on teaching is an interrelated set of beliefs and intentions that gives direction and justification to our actions. It is a lens through which we view our work as educators. We may not be aware of our perspective because it is something we look through, rather than at, when teaching. Each of the perspectives is a unique blend of beliefs, intentions, and actions…".^4^ For the purpose of this paper conceptions and perspectives are used interchangeably. Pratt^5^ has categorized five perspectives of teaching: transmission, apprenticeship, developmental, nurturing and social reform. In a report where 1000 responses were analyzed using the Teaching Perspectives Inventory, it was shown that teachers of adults who were starting in their careers or who were still in training had higher nurturing scores, whereas teachers whose trainees were older, had lower nurturing scores.^6^ This suggests that conceptions of teaching are dynamic, indicating a need to know the conceptions of the teaching of teachers in different stages of their careers, or in different levels of education. Most teachers have one to two perspectives as their prevailing view.^4^ Trigwell, Prosser and Waterhouse^7^ were the first to report that there was a relation between the teachers' approaches to teaching and the students' approaches to learning. Teachers who were teacher-centered and mainly used transmission-type teaching formats tended to inflict a surface approach to learning by the learners. This and other studies^7^^,^^8^^,^^9^ indicate that conceptions of teaching impact the interaction between the teachers and the learners. In any setting of education, the challenge is to understand how conceptions of teaching can be used positively to reinforce a learning attitude in the students or trainees.

In undergraduate medical education, recognizing the conceptions of teaching and learning of faculty can promote reflections of teachers and stimulate changes in the teaching behavior. In addition, it can pave the way for faculty development as needed by the faculty, aligned with their institutions' directions.^10^ Jacobs and colleagues^10^ developed an instrument, the Conceptions on Learning and Teaching (COLT), that measured the conceptions of learning and teaching of teachers in student-centered undergraduate medical education. COLT is a 3 factor, 18 item instrument. Using the COLT, two medical institutions were compared, one with a strong tradition of student-centered curricula and one which recently shifted to student-centered curricula. The teachers in the institution with a stronger tradition on student-centered education gave higher scores on the factors appreciation of active learning and orientation to professional practice. These factors were known to support student-centered education, indicating that the teachers' perceptions were influenced by exposure to the institution's student centered-curricula.^10^ Studying conceptions of teaching in postgraduate medical education is more complex. As rightly noted, an important part of medical education takes place in clinical settings, with unique challenges to teaching.^11^ In the clinical settings, the circumstances are such that educators cannot control the cases one will encounter and trainees have different levels of competence.^11^ In medical education, expertise in a particular field, e.g., cardiology is considered equivalent to being a qualified clinical teacher.^11^^,^^12^ In addition, the development of medical teachers is influenced by the changes they go through in becoming a clinician^13^ and past conceptions of self as a student influence their current expectations of their trainees.^14^ According to MacDougall and Drummond,^13^ medical teachers learn by observing other teachers, and once they grasp what they have seen, they practice these teaching skills on the younger medical trainees. It is a common observation that clinical teachers rarely receive formal instruction in teaching. How clinical teachers form their conceptions of teaching is less understood, nor what would be pertinent factors that influence a clinical teacher's own conception of teaching. In addition, it is relevant to understand how much of a clinical teacher's conceptions of teaching is brought about by past experiences as a learner and how much is brought about by observing other medical professionals. Since in higher education and undergraduate medical education, it has been shown that conceptions influence how students or trainees respond to their teachers, it is pertinent to determine if the same is true in postgraduate medical education. Given these findings, our research question was: how do the conceptions of the teaching of a postgraduate clinical teacher impact their training of residents?

## Methods

### Setting

The study was conducted at De La Salle University Medical Center (DLSUMC), a teaching hospital based in the province of Cavite, which is located 50 kilometers from Manila, the Philippines. DLSUMC is the teaching hospital of the De La Salle University College of Medicine. The majority of the doctors who practice here are alumni of the college, the majority also are members of the faculty of the college of medicine and at the same time are involved in the residency training of their different departments. In the Philippines, the residency training duration for pediatrics and internal medicine are three years, and for residencies requiring surgical skills, four to five years.

### Study design and participants

We used a combined data collection,  consisting of a survey followed by interviews. For the survey, we used the Conceptions on Teaching and Learning of Teachers (COLT) instrument, which is designed to measure the conceptions of teaching. We invited physicians practicing at De La Salle University Medical Center, a teaching hospital to fill out the COLT. Thereafter individual interviews using a semi-structured questionnaire with vignettes were done.  The vignettes briefly summarized the five perspectives of teaching according to Pratt and Smulders.^5^ The qualitative part allowed us to complement results from the survey, utilizing a convenient sampling of clinical faculty who previously filled out the COLT instrument. Ethical review was done by the Independent Ethics Committee of the De La Salle Medical and Health Sciences Institute.  The participants were informed of the purpose of the study in writing and orally and were assured that participation was purely voluntary.

###  

### Data collection

#### Survey

For the survey, an invitation was sent out to the clinics of the 292 clinicians practicing in the hospital, belonging to different clinical departments between June-October 2017. One medical staff meeting was also used as an occasion to invite medical staff to fill out the COLT. COLT is an 18-item questionnaire with a five-point Likert scale (1=strongly disagree, and 5=strongly agree), see Appendix 1. Since COLT was originally designed for undergraduate medical institutions in the Netherlands^10^ with student-centered curricula, we invited four Filipino physicians who are both involved in undergraduate medical education and postgraduate training to evaluate the original COLT to make it more appropriate in the context of a postgraduate environment. Minor revisions were done, and it was agreed that the content of COLT was applicable to the setting of postgraduate residency training. These revisions included replacing the word "students" in the original COLT to "residents" and modifying item number one which originally was phrased as "Students should first master basic science knowledge before they can formulate their own goals" to "Residents should first master general medical principles before they can formulate their own learning goals". The first author was responsible for the data collection.

#### Interviews

After the survey, a purposive sample of twelve clinicians from the respondents of the survey was invited to participate in the interview. These clinicians represented a wide spectrum of experience as a doctor, fields in medicine, age and administrative positions. They all agreed to take part. These physicians came from different specialties which included internal medicine, pediatrics, anesthesia, and surgery. Different fields of internal medicine were also represented, such as pulmonary medicine, endocrinology, nephrology and infectious diseases. Some of the participants hold or previously held administrative positions. A semi-structured questionnaire was used, containing vignettes to introduce five different perspectives and to highlight the different features of the different perspectives of teaching as described by Pratt,^5^ see Appendix 2. The interviews were done by the first author. The duration of the interviews were from 25-45 minutes each. The interviews were recorded and transcribed verbatim.

### Data analysis

#### Survey

We performed a confirmatory factor analysis (CFA) to determine if the Filipino data fitted the 3- factor structure of the original 18-item COLT.^10^ We used the following criteria and associated pre-determined cut-off values to gauge goodness of fit: Tucker-Lewis index (TLI > 0.9), comparative fit index (CFI>0.9), root mean square error of approximation (RMSEA<0.08) and standardized root mean square residual (SRMR<0.08). In the present study, a sufficient fit was deemed to have been achieved when 4 criteria produced significant results.

To identify different teacher profiles, cluster analysis was done, where objects were grouped together because of their shared similarities. We used a K-means cluster analysis on the complete data using the three original subscales in Jacobs and colleagues.^10^ R version 3.6.0 was used for the analyses.^15^ For the CFA we applied R package Lavaan.^16^

#### Interviews

The interviews were analyzed through template analysis.^17^ We started with a priori codes (see Appendix 3), and subsequently, a series of coding patterns containing structured themes of different tiers were then applied to the data. Interviews continued until theme saturation was reached. Theme saturation was defined as the occurrence of recurring themes, and there was already adequate data to support a theme. Independent analysis of the first three transcripts using the a priori template was done by JLP and SH. This gave rise to the initial template, which was used by JLP and SH for coding transcripts 4-9. The final template was used by coding interviews 10-12. Further discussions to reach consensus on the themes that were emerging were done after each step.

#### Reflexivity

Being aware of the effects of the researchers in the data collection and analysis and subsequent creation of concepts and knowledge, the background of the different authors are as follows: JLP is a practising internist and cardiologist and has been a faculty in the college of medicine for many years. He was a former chair of the department of IM. JD is a psychometrician at the School of Health Education of Maastricht University. JJ is a medical doctor and an educationalist. SH is a health scientist with an educational background. CvdV has training in psychology and psychometrics with many years of engagement in medical education and medical education research.

## Results

### Survey

The response rate was 59% with 172 respondents of the COLT included in the analysis. Ninety-three were females, and 79 were males, the majority of the participants belonged to age group 30-55 years. A CFA was performed on the data using the three original subscales in Jacobs and colleagues.^10^ Before CFA, two influential outliers were removed, and six missing answers were imputed using the means. The results of the CFA showed a suboptimal fit; four measures (SRMR = 0.090, RMSEA = 0.082, CFI = 0.758 and TLI = 0.720) were outside the range for adequate fit as indicated by Jacobs and colleagues.^10^

**Table 1:** Modified COLT Questionnaire and scores based on the Filipino Data

Item		Overall score (mean ± sd)
Factor 1. Teacher – Centeredness		
1	Residents should first master general medical principles before they can formulate their own learning goals.	4.21 ± 0.91
2	Residents learn best when the learning process is guided by an expert who has an overview of the field of interest	4.67 ± 0.60
3	When residents discuss a topic without an expert being present, they do not know at the end of the session if the questions have been answered correctly.	3.99 ± 0.88
4	As a teacher I have to indicate clearly what is important and what is less important for the residents to know.	4.2 ± 0.77
Factor 2. Appreciation of Active Learning		
5	Residents learn a great deal by explaining the subject matter to each other.	4.16 ± 0.64
6	Learning materials and teaching should invite residents to come up with examples to illustrate the subject matter.	4.37 ± 0.54
7	I think it is more important for residents to be able to analyze and critically appraise the subject matter than to memorize facts	4.7 ± 0.57
8	I think it is important that residents advise each other about the best ways to study.	3.95 ± 0.73
Factor 3. Orientation to Professional Practice		
9	Being introduced to the day-to-day practice of their future profession motivates residents to learn.	4.39 ± 0.57
10	It is a good learning outcome when residents demonstrate that they can apply their knowledge during their activities in situations in professional practice.	4.59 ± 0.50
11	I think that interactions between the residents and me are an important aspect of my teaching	4.66 ± 0.50
12	Discussing topics with each other helps residents learn how to deal with different points of view, so as to gain a deeper understanding.	4.62 ± 0.51

CMIN/DF is 2.15, which is within the range (<3), but p =0.001. Maximum correlation between residuals is 0.272, which is far above 0.1.  Removing 6 items (2,5,7,8,11, and 14) improved the fit to an acceptable level since four criteria of goodness of fit reached a sufficient level (SRMR = 0.058, RMSEA = 0.050; p=0.48, CFI = 0.936 and TLI = 0.918). Maximum correlation between residuals dropped to 0.174. The Cronbach's alpha for Teacher Centeredness, Appreciation of Active Learning and Orientation to Professional is 0.53, 0.51 and 0.75, respectively. Table 1 shows the modified COLT and the overall scores of the results of the survey.

### Cluster analysis

A K-means clustering was performed on the complete data using the three original subscales in Jacobs and colleagues.^10^ The optimal number of clusters was unclear since elbow, gap and silhouette methods disagreed or indicated no clusters. The visual inspection resulted in the choice for a three-cluster model. We used the score for teacher centeredness (TC) factor as the basis for the clustering, similar to what Jacobs and colleagues did.^18^ Fifty-one percent belonged to cluster one which we refer to as transmitters, because of the high score given to the factor TC. Majority of those belonging to cluster 1 are females, and it also has the youngest age group, 44.9 years being the mean age. Cluster 2, which was 37% of the group, had the highest mean age with more males. The properties of cluster 2 in reference to the factor TC was in between clusters 1 and 3 (see Table 2). We call cluster 2 as the intermediates. The score of cluster 2 was lowest among the 3 clusters in the factors of active learning (AL) and professional practice (PP). Cluster 2 seems to be in transition, low in support for the factors supporting student centeredness with a relatively high score for TC next to cluster 1. Cluster 3, which we call the nurturers, represents 12.2% of the respondents. Among the 3 clusters, this group has the longest number of years in practice, 13.2 years. It also has the lowest preference for TC items in the COLT. This implies that the longer serving faculty seemed to less favor factor TC, which points to a shift in their conception as they grow older or have served for a longer period as faculty.

**Table 2:** Properties of the clusters

Cluster	Count	TC	AL	PP	Age	Gender	Marital	NYP
1	88	4.49	4.52	4.84	44.92	0.38	0.75	10.89
2	63	4.29	3.98	4.17	46.25	0.57	0.84	11.62
3	21	3.26	4.32	4.56	46.00	0.48	0.52	13.21

Mean values per cluster (TL = teacher-centered, AL = active-learning, PP = professional practice). Gender = proportion males, Marital = proportion not single, NYP = average number of years in professional practice

### Interviews

The analysis is presented in 3 parts: the initial response to a question about being an effective clinical teacher, the perceptions on clinical teaching after the presentation of the vignettes and the factors that were considered helpful or hurdles in changes in teaching style.

The first intuitive response of clinical faculty of an effective clinical teacher was somebody who is able to transfer knowledge in a way that is easy for the trainees to understand.

When presented with the vignettes that described the five perspectives the clinical teachers could identify with the practices and features of the five perspectives and indicated their use in practice. The analysis gave insight into the factors that were helpful of perceived hurdles in the implementation of a teaching style.

### 1. The first intuitive responses

The first intuitive responses on the beliefs of what an effective teacher should be indicated that clinical faculty's understanding of clinical teaching was formed by their prior experiences as a learner and by 'common' beliefs on teaching. A common notion of what an effective teacher should be was indicated as being able to simplify the delivery of learning material and makes it easier for the trainees to understand and to apply the concept in their clinical rotation.

"simplify the topic, whatever clinical topic, so the residents will be able to understand it" (No 2, F)

"the ultimate goal will be for that learner to apply whatever you have taught him. So in the clinics, you would see him perform better, and he would have a better clinical judgment of cases seen" (No 7, M)

### 2. Recognition in practice and preferred style

When asked about their perspectives as a clinical teacher after reading the vignettes (see Appendix 2), two perspectives emerged. Role modeling or apprenticeship was the most common perspective chosen by the clinical faculty, followed by the nurturing perspective. Furthermore, after reading the vignettes, their perceptions were more nuanced, it was indicated that although initially unaware of the perspectives, the interviewees also practiced features of other perspectives, e.g. features of the nurturing and developmental perspective were recognized in their own teaching style.

"as much as possible, I do not want to discourage them or put them down. …if they commit a mistake, I want them to reflect on that mistake…meaning to realize the reason why a mistake happened or what will they do the next time it happens and why it happened, in the first place..." (No 10, M)

 In addition, the role modeling or apprenticeship perspective was connected to experiences as a student and a medical trainee.

"It is really applicable in our setting, and it is best learned when you apply it in practice because for me, I learned a lot, when I was a clerk when I was practicing what I learned from the didactics from the first year to the third year...residency and fellowship training, it was really there that I was able to apply and practice the ones that I learned during medical school. That is for me as a student. As a teacher, I think modelling and coaching, that is the only way that you can teach a student. It is like being a parent because you have to be a role model... the kids will just copy what you do." (No 4, F)

The vignettes were also recognized as opportunities in teaching that were more consistent with a vision on their role as clinical teachers:

"I think…because it is more relaxed, no 3 (nurturing) and no. 4 (developmental), it is a friendlier way to let these trainees learn, and there are different types of trainees like what was said…there are enthusiastic learners, and there are average learners…so you really have to adapt to them...what they know and what their skills are…" (No 1, M)

### 3. Factors associated with changes in teaching style

Changes in teaching style had been prompted by several factors, namely: personal experiences, the institution itself, maturity of the teachers and global evolution in medical education.

Although trained in a different way during their early years as medical professionals, a change in teaching style was prompted by prolonged experience as a teacher and by reflections on their experiences as a teacher. Interviewees indicated to have broadened the topics they wanted trainees to learn or pay attention to. Whereas when they started as teachers, their emphasis was on patient care alone, through the years other needs of the patients were included, such as the overall welfare and experience of the patients.

"During the first years of clinical teaching I was more really into teaching; As a new graduate then I was asking the trainees the basic knowledge, for example, asking the resident about pathophysiology and management but as you grew mature and grew older in the practice you start paying attention to the other aspects like patient care, patient safety, administrative or the people around the hospital." (No 10, M)

The institution was instrumental in facilitating these changes. The culture and traditions in the institution motivated to change their perspectives. This was especially true for those clinicians who graduated from other medical institutions and could experience the difference between their old institution and the present institution. In addition, changes in the individual context mattered, e.g., the maturation in their personal responsibilities, in becoming senior faculty, or changes in their personal lives.

"At the start, when I was younger, I just wanted to teach them, and tell them what I know. It was like I was trying to prove something. That I know something. Later on, eventually, especially when my kids grew up, I started to be influenced by the thought -- how I would interact with them if they were my children. Maybe that is why I can talk to them differently now since years earlier, my children were smaller, and now my children are bigger, and I interact with them differently. So I look at them in the same way I try to interact with my children." (No 4, F)

And lastly, the evolution in medical education – it was acknowledged that the global innovations in medical education, such as improving the learning environment, were instrumental in priming the changes in their perspectives on teaching.

### 4. Perceived hurdles or barriers for changes in teaching style

There are several factors that were identified that could obstruct the implementation of teaching styles that were preferred. It was acknowledged that efforts to implement teaching styles that are "ideal" could be impeded by the context and the learners themselves. For the context, colleagues and especially older faculty could intentionally or unintentionally impede initiatives by the younger faulty. The stature that senior faculty have gained became an important barrier because these senior faculty were very influential and could steer how other faculty think about almost any aspect related to being a faculty, including conceptions of teaching.

 "Most of them came ahead of us, who have been indoctrinated into the traditional way of doing things, and for them if it worked then why should I change it?" (No 5, M)

With regard to the role of the institution, although the institution itself could create a learning environment that supported the teaching style, in other aspects e.g. technology that could support the clinical training, it could fall short of the expectations. In addition, the competence of learners was an important variable too– since the institution can only choose from among those who applied to the training programs of the different departments, there may be a wide range in terms of aptitude and knowledge among the trainees and this could be a hurdle to adopt a certain, preferred teaching style. This is not limited to the aptitude but also includes the attitude of the trainees. And lastly, not surprisingly, time was often mentioned as a hurdle for change. The most common issue was that teaching competed with their clinical work. This was also caused by the current setup in the institution where doctors earn from their private practice.

"it affects our clinical practice so the most important is time. We are sacrificing our practice and income when we spend time to teach. Also the residents follow a structured schedule, for example they have endorsement time or conferences so even if I am free I could not meet them. So I really have to allot time to teach." (No 7, M)

## Discussion

For the first part of this study, the survey, we have shown that the COLT although initially designed for undergraduate medical setting in the Netherlands, was a valid tool in a different setting and different population, with some modifications. Furthermore, in this Asian context, 3 clusters were evident, with 51% of the participants belonging to the teacher-centered or transmission perspective. In the interview study, we have shown that postgraduate faculty have strong opinions on what is an effective teacher and how to carry this out and most are based on their previous experiences as students or trainees. The data from the interviews also supported our result of the survey, where a majority of the participants initially chose the transmission perspective as the perspective which best represented their conception of teaching. However, when introduced to the other perspectives, the apprenticeship and nurturing perspective were recognized, in spite of the fact that almost all of them were exposed to a teacher-centered or transmission perspective as students. The longer serving faculty were less in favor of the transmission perspective. Our findings showed that exposure to other perspectives such as nurturing, developmental and social reform, initiated a recognition as well as an expressed interest in the other perspectives, which provided opportunities in teaching that was more consistent with perceived visions on their own role as teachers. Perspectives of teaching that take into consideration the well-being of the trainees were clearly preferred.

There are several potential uses and applications of measuring conception of teachers, in this case particularly, in the postgraduate setting. A tool to measure conceptions can stimulate reflective thinking on the perspectives of teachers.^10^^,^^19^ Reflecting on these perspectives can convert a tacit pedagogic knowledge to explicit, which can improve the teaching effectiveness of a medical teacher.^12^ It can be a stepping stone and aid in designing appropriate faculty development programs.^10^ As emphasized by Collins and Pratt there is more than one acceptable way of teaching; thus, peoples' expressed perspectives are a robust mechanism to stimulate or promote faculty development exercises.^19^ It is a common observation that individuals have one dominant perspective and 1-2 supporting perspectives, understanding why one perspective is more dominant is a way to understand teachers on what is a perspective makes it close to their values and personal philosophies than other perspectives.^5^ Calkins, Johnson, and Light^20^ believe that helping faculty develop a complex understanding of teaching is a crucial component in a faculty member's professional development which is equivalent to their being able to develop their research and clinical skills. These findings are in line with a study that showed that faculty conception of teaching did not depend on their teaching context, duration of teaching, or their specific disciplines, suggesting that any faculty member can develop alternative conception overtime when introduced to different conceptions of teaching.^20^

Additionally, our results showed that faculty who have been teaching for a longer period of the time chose to nurturing as their preferred perspective. This was also supported by the quantitative data in the 3 clusters, which showed that the older faculty gave low scores to the factor TC compared to the younger faculty. In a report involving 116, 621 teachers who answered Teaching Perspectives Inventory, which used the same classification as the five vignettes presented to our participants and which is available online, nurturing is the most commonly chosen perspective, followed by apprenticeship.^19^ We think that in postgraduate setting, measuring the perceptions of clinical teachers can promote reflective thinking and a discussion of the outcomes within the teaching staff could improve the quality of training and the training environment itself.

Pedagogical literature provides evidence that a student or learner-centered perspective has many advantages and benefits.^22^^, ^^23^ Wright in a review^24^ concluded that many college teachers believe that a student-centered setting leads to a more effective learning environment. Mclean and Gibbs^25^ note that in the past decades there had been a major change in medical education, one of these is the rise of student-centered learning or learner–centered education wherein the focus has shifted from teachers as transmitters of knowledge to the students being the center of the learning experience, which has shifted the roles of teachers to being facilitators. Lea, Stephenson and Troy^21^ assert that interest in student-centered learning is well established in all levels of education, but a major problem related to theoretical and applied research in this area is definition. They clarified that student-centered learning means: reliance on active learning rather than passive, an emphasis on deep rather than superficial learning, a greater sense of autonomy among learners, more active role in terms of responsibility and accountability on the part of the students, interdependence between the students and the tutor, mutual respect between the teacher and learner and a reflexive approach to the teaching and learning process. Student-centered education seems to be more appropriate in medical education since people who choose a medical career place a high premium on personal autonomy.^26^ However, the problem with these propositions is that it tends to relegate other perspectives of teaching to a lower rank, especially teacher-centered perspective. We agree with Pratt and colleagues,^5^ who challenge that there is sufficient evidence to state that there is no single way to teach adults. Rather, what is needed is what he calls "a plurality of perspectives on teaching that recognize and respect a diversity of teachers, learners, content, context, ideals, and purposes".^5^ For them, the analogy is: until one experiences a different culture, the culture one grew up with is invisible to that person, in the same way, other ways of teaching are invisible to a teacher if he or she knows only one perspective of teaching. Different perspectives are best suited to a specific population of students, goals, values and contexts. Lastly, it is superficial to assume that the transmission perspective is the same as lecturing.^5^ Clinical faculty must have the opportunity to discuss the different perspectives and their own perspective as a way to improve their teaching by learning from the experiences and perspectives of their colleagues.

### Limitations

A potential limitation of the current study is that some of the participants may have a strong opinion about postgraduate training; thus, they agreed to be a part of the study. On the other hand, our participants had a good representation in terms of age and experience as a faculty. We did not consider it a constraint having used COLT to initiate a discussion on perspectives of clinical teachers on learning in our setting, even though it was designed for a different population.

## Conclusions

Our study has shown that the COLT although initially designed for undergraduate medical setting in the Netherlands, was a valid tool in a different setting and different population, with some modifications. Both the survey and the interview study showed that majority of the faculty chose the transmission perspective initially, but when introduced to the other perspectives, preferred apprenticeship and nurturing. The faculty readily embraced other perspectives of teaching that they believe in taking into consideration the well-being of the trainees. Additionally, the longer-serving faculty were less in favor of the transmission perspective.

There are several potential uses and applications of measuring conception of teachers, in this case particularly, in the postgraduate setting. It can be a tool to stimulate reflective thinking on the perspectives of teachers. Reflecting on these perspectives can improve the teaching effectiveness of a medical teacher. It can be a stepping stone and aid in designing appropriate faculty development programs. As emphasized by Pratt^5^ there is more than one acceptable way of teaching; thus peoples' expressed perspectives can be used to stimulate productive discourse on faculty development.

### Conflict of Interest

The authors declare that they have no conflict of interest.

## Supplementary material

Supplementary file 1 Appendix 1. COLT Questionnaire as presented to participants

Supplementary file 2 Appendix 2. Interview Guide

Supplementary file 3 Appendix 3. A Priori Codes
