# Supplementary material for: Understanding teaching and learning conceptions among clinical faculty as a means to improve postgraduate training
Source: Int J Med Educ. 2020 Aug 28;11:175–85. doi: 10.5116/ijme.5f2a.76eb (PMC7882127; doi:10.5116/ijme.5f2a.76eb)
Supplement: Supplementary file 1 — Appendix 1. COLT Questionnaire as presented to participants [file ijme-11-175-S1.pdf]

Appendix 1.

COLT Questionnaire as presented to participants

Factor 1- Teacher Centeredness

1. Residents should first master general medical principles before they can formulate their own learning goals.
2. I think that in small group learning, the clinical faculty determines what the residents should learn instead of the residents determining their own learning goals.
3. Residents learn best when the learning process is guided by an expert who has an overview of the field of interest.
4. When residents discuss a topic without an expert being present, they do not know at the end of the session if the questions have been answered correctly.
5. There is a logical sequence to learning.
6. As a teacher I have to indicate clearly what is important and what is less important for the residents to know.
7. I think that as an expert in my field I am eminently suitable to transmit my knowledge to residents and that residents should not have to look up that knowledge for themselves.
8. When residents collaborate they teach other the wrong things.

Factor 2 - Appreciation of Active Learning

9. Residents learn a great deal by explaining the subject matter to each other.
10. Learning materials and teaching should invite residents to come up with examples to illustrate the subject matter.
11. Small group learning motivates residents to study.
12. I think it is more important for residents to be able to analyze and critically appraise the subject matter than to memorize facts.
13. I think it is important that residents advise each other about the best ways to study.

Factor 3 - Orientation to Professional practice

14. I think it is important that educational assignments are derived as much as possible from the residents' future professional practice.
15. Being introduced to the day-to-day practice of their future profession motivates residents to learn.
16. It is good learning outcome when residents demonstrate that they can apply their knowledge during their activities in situations in professional practice.
17. I think that interactions between me and the residents are an important aspect of my teaching.
18. Discussing topics with each other helps residents learn how to deal with different points of view, so as to gain a deeper understanding.
