# Supplementary material for: Understanding teaching and learning conceptions among clinical faculty as a means to improve postgraduate training
Source: Int J Med Educ. 2020 Aug 28;11:175–85. doi: 10.5116/ijme.5f2a.76eb (PMC7882127; doi:10.5116/ijme.5f2a.76eb)
Supplement: Supplementary file 2 — Appendix 2. Interview Guide [file ijme-11-175-S2.pdf]

Appendix 2.

Interview Guide

Semi-structured Questionnaire

1. What is an effective clinical teacher to you?
2. Which perspective (conception) do you identify most with?

Vignettes

1. The central belief in this perspective is that teaching is, primarily, a matter of effective delivery of content. The primary responsibility of the teacher is to present the content and then help learners accurately reproduce that same content in its authorized and accurate forms. The roles of a teacher are that of a presenter, expert and authority. What is learned should closely resemble what was taught. Assessment therefore is concerned with reproducing information or procedures according to standards.

2. The central belief in this perspective is that expert knowledge is best learned in contexts of application and practice. The central task of a teacher within this perspective is to help the learners do what they can on their own, under the guidance of a more experienced teacher or in collaboration with more capable peers, in other words the teachers serve as role model and coach. Assessment is linked to authentic tasks, and within the contextual standards.

3. The central beliefs are that learning is related to a learner's efficacy and that the learning environment should be safe, trusting and comfortable enough for learners to take risks, make mistakes and celebrate success. The goal of this perspective is to help people become more confident and self-sufficient learners. The primary responsibilities of a teacher include promoting and supporting learner's self-efficacy while enabling greater responsibility for learning. Assessment strategies include emphasizing frequent low-stakes feedback, rather than high-stakes (grades) to encourage and support learning, and allowing learners to share in the responsibility for the development of assessment of their learning.

4. The key belief in this perspective is that the key to learning and teaching lies in discovering what the learner already knows or believes (prior knowledge) and building effective "bridges" between present and desired ways of thinking. The teacher's role is that of a guide and co-inquirer. Responsibilities of the teacher include challenging students' understanding of content, encouraging new ways of thinking and reasoning and promoting deep approaches to learning. Assessment strategies include avoiding a surface approach to assessment, looking beyond answers for underlying reasoning, and looking for use of different forms of reasoning.

5. This perspective is distinctive for the presence of an explicit, well–articulated ideal or set of principle that are linked to an explicit vision of a better society. The role of a teacher includes being an advocate for an ideal, and a model for challenging authoritative positions. Responsibilities of a teacher include clarifying relevance of ideal in classroom, discipline, work/practice and society and demonstrating relationship and connection between ideal and context. Assessment strategies include emphasis on critical thinking and recognizing conventional standards of evaluation while seeking to transcend externally imposed limits.

3. Can you reflect on the significance for your teaching style?
4. Has there been a change in your perspective since you started as a teacher, why or how did it happen?
5. What are the barriers or obstacles that you have experienced in implementing your perspectives in teaching?
