# Supplementary material for: Understanding teaching and learning conceptions among clinical faculty as a means to improve postgraduate training
Source: Int J Med Educ. 2020 Aug 28;11:175–85. doi: 10.5116/ijme.5f2a.76eb (PMC7882127; doi:10.5116/ijme.5f2a.76eb)
Supplement: Supplementary file 3 — Appendix 3. A Priori Codes [file ijme-11-175-S3.pdf]

Appendix 3.

A Priori Codes

A. A good teacher

1. Transmits knowledge and skills within a time frame
2. Flexible and able to adjust to different types of trainees
3. Role model

B. Expectations of a clinical teacher of their trainees

1. trainees to be competent
2. apply what their consultants taught
3. prepare for exams

C. Perspectives

1. Old and new styles of teaching
2. Nurturing and developmental - "learner friendly"
3. Transmission too stiff
4. Pro-resident
5. Teach in such a way trainees are motivated to teach their juniors
6. Consultants can learn from residents

D. Barriers for clinicians to implement their perspectives

1. time
2. money
3. different quality of learners
